# Gingivectomy as a Treatment for Orthodontic-Induced Gingival Enlargement

**DOI:** 10.7759/cureus.67069

**Published:** 2024-08-17

**Authors:** Komal Agrawal, Unnati Shirbhate, Priyanka Paul, Prachi Khandelwal

**Affiliations:** 1 Periodontics, Sharad Pawar Dental College and Hospital, Datta Meghe Institute of Higher Education and Research, Wardha, IND; 2 Public Health Dentistry, Sharad Pawar Dental College and Hospital, Datta Meghe Institute of Higher Education and Research, Wardha, IND; 3 Dentistry, Sharad Pawar Dental College and Hospital, Datta Meghe Institute of Higher Education and Research, Wardha, IND

**Keywords:** periopack, gingiva, orthodontic treatment, gingivectomy, external bevel gingivectomy

## Abstract

The side effects of an antipsychotic drug, such as fibrous overgrowth and gingival inflammation, or a combination of both, can lead to gingival enlargement. Causes for developing plaque include neglected cleanliness, architectural differences disturbing contact, faulty restorative buildup, cavities, and orthodontic appliances. Hence, in actual clinical scenarios, finding out the exact reason with precision is the key to appropriate therapeutic intervention. The presented clinical case is about a 29-year-old female patient who was referred to the Department of Periodontics due to a swollen gums complaint. The drug administration was first done, with the second step constituting surgical reduction of excessive gingival tissue under local anesthesia using gingivectomy. After the surgery, an application of GC Coe-Pack (GC America Inc., USA) was made that acted as a dressing for the tissue and promoted healing. Follow-up was done to assess the patient’s gingival and periodontal conditions as requested through recall. In the post-procedure circumstances following that, the ideal gingival height was reached. All the results were healthy in the given case presentation with no remaining supra bony pockets, achieving natural-looking gingival architecture, thus enhancing esthetics and decreasing plaque accumulation. The interventions of surgical gingivectomy can be deemed effective in this case.

## Introduction

Gingival enlargement, often referred to as gingival hyperplasia, is a prevalent sign of numerous disorders, including systemic and drug-induced inflammation, pregnancy, and leukemia, along with problems generated by orthodontic therapy [1]. Hence, gingival enlargement may result from fibrous hyperplasia and inflammation, or either one, based on the extent of fibrosis as well as inflammation. Other reasons that may be linked to gingival enlargement include hormone fluctuations, vitamin deficiencies, pharmaceutical effects, genetic, random, mild trauma, and systematic disorders [2]. Therefore, a reliable identification of the enlargement source is essential to the treatment procedure [3]. Individuals having fixed orthodontic treatment for longer than 12 months are at risk for developing periodontal disorders [2,4]. Gingival hyperplasia or overgrowth is a particularly frequent side-effect associated with fixed orthodontic appliances [5]. The orthodontic bands' nickel ions, which produce fibroblastic growth, are the source of the gingival expansion brought on by this treatment. The cause of this is that nickel ions are used to make orthodontic brackets. Among the many interactions within nickel ions along with orthodontic bands, one of the most significant is the interaction between the nickel ions and the gingival tissues involved in the method, resulting in fibroblast proliferation along with gingival overgrowth [1,6]. One of the effects of this growth is the incapacity to maintain good oral hygiene. Plaque accumulation on teeth with inadequate oral hygiene practices is the main cause of gingival expansion [7]. There is currently evidence linking certain orthodontic individuals' bacterial dental plaque to periodontal issues [5]. This is a significant contribution to guaranteeing that every patient wishing to receive orthodontic therapy has a thorough examination of their periodontal health [1]. There is a need to instruct the patient about personal oral health care, the importance of eradicating risk factors, and the importance of maintaining hygiene and oral health. Gingivectomy is a method of surgical treatment of excessive growth of the gingival tissue. This operation is suitable for patients with gingival hyperplasia, persistent periodontitis, or those who expect a better appearance [4]. This case report presents photographic documentation and a description of the gingivectomy dental procedure through the steps of pre-operation assessment, surgical technique, post-operation management, and a summary of the patient’s result. The aim is to enhance awareness of periodontal surgery in dental practice by implementing the concerned clinical features, challenges, and results concerning gingivectomy [8].

## Case presentation

**Figure 1:**
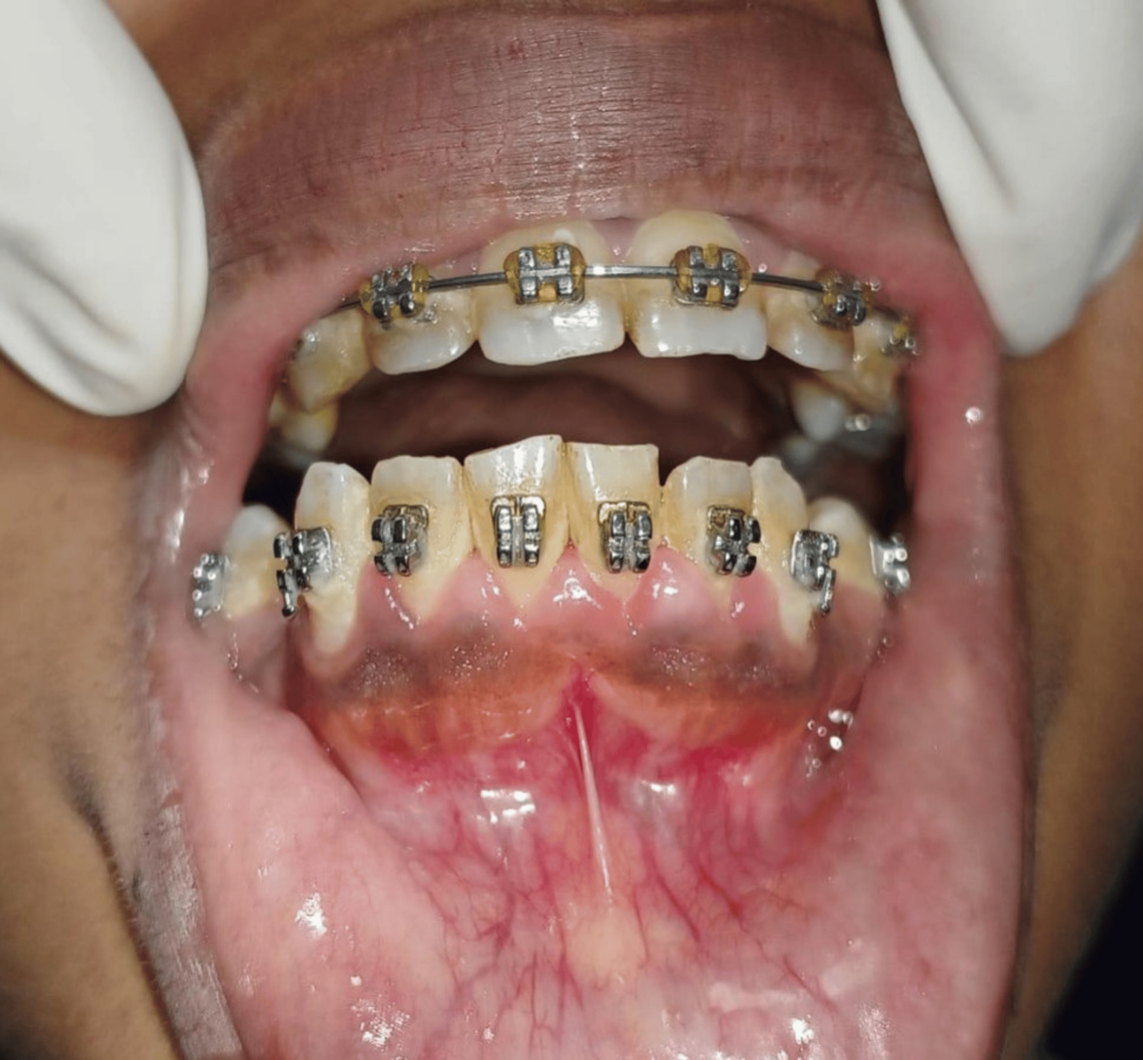
Preoperative image of patient undergoing orthodontic treatment. Image credit: Komal Agrawal

A 29-year-old female patient undergoing orthodontic treatment for one year was referred to the Department of Periodontics with a complaint of swollen gums. Clinically, there was marginal and interdental gingival enlargement in the lower anterior region (Figure 1).

**Figure 2:**
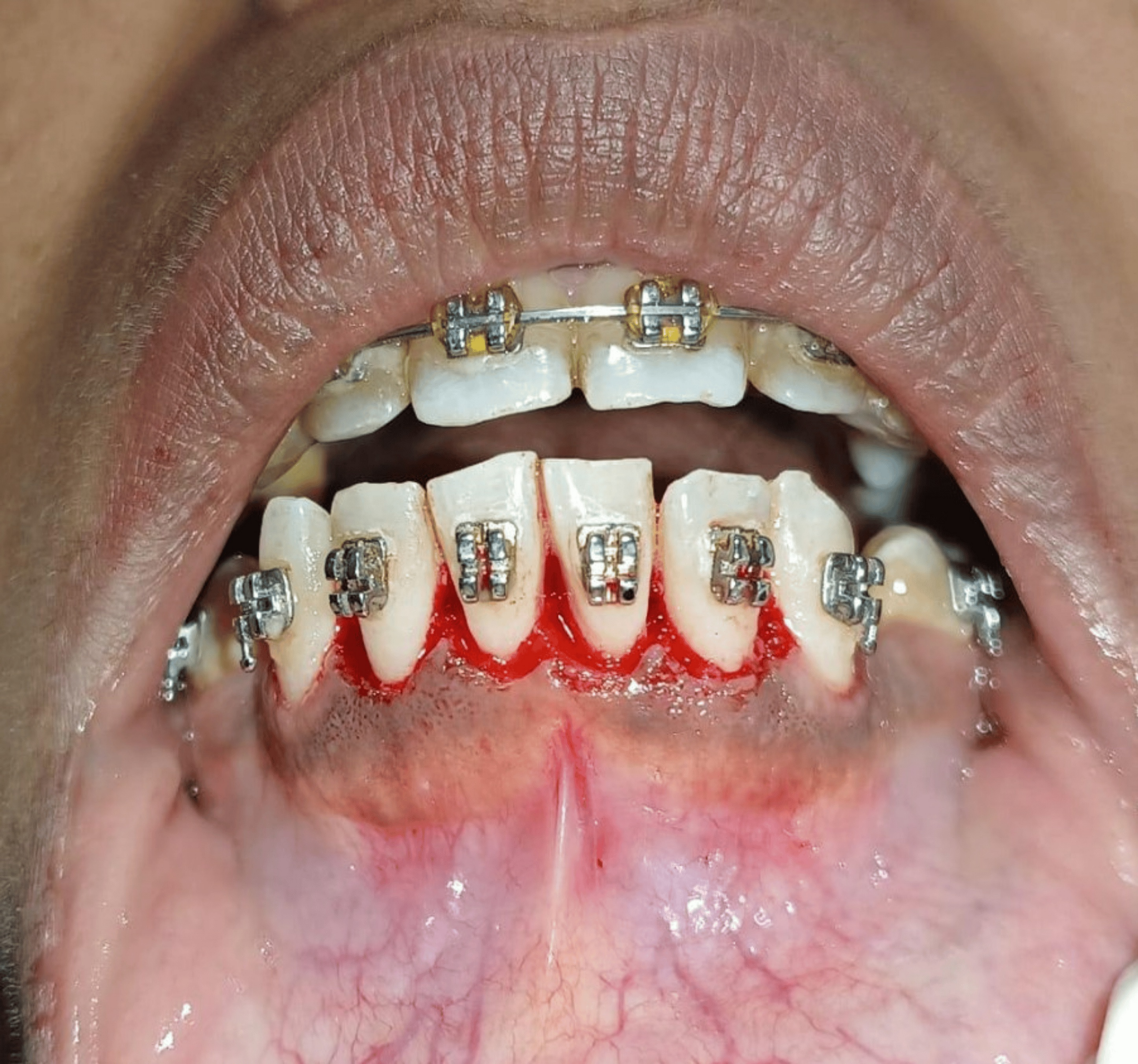
Gingivectomy performed using the conventional scalpel technique. Image credit: Komal Agrawal

**Figure 3:**
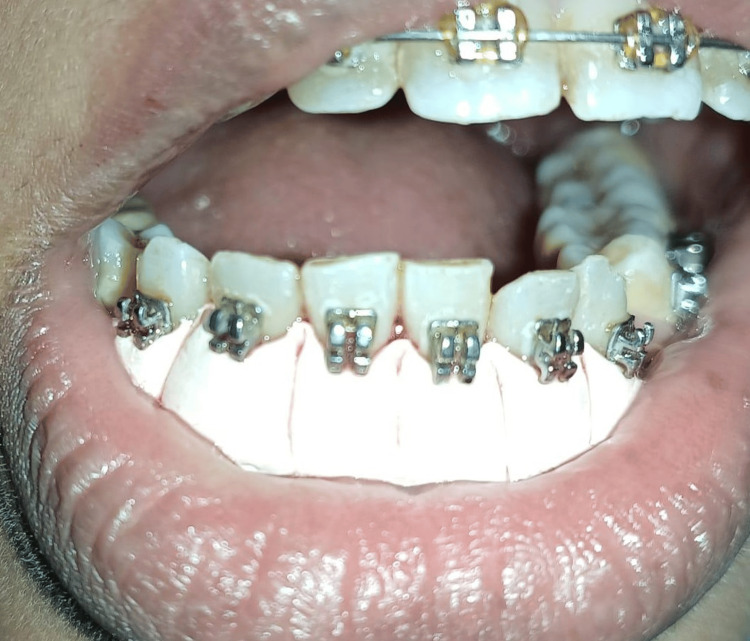
Periodontal pack administered after the surgery. The periodontal pack GC Coe-Pack (GC America Inc., USA) was given. Image credit: Komal Agrawal

The general examination found that the patient had no contributing medical history. Examination revealed extensive gingival tissue and pseudo pockets associated with pale pink edematous gingival characteristics. Calculus and plaque were seen on each tooth. Based on the findings, the patient was advised to initial therapy followed by surgical intervention along with hematological evaluation. Firstly, supragingival scaling and polishing were performed. The patient was instructed to use a soft toothbrush and perform gentle brushing using the modified bass technique. All the hematological tests were done, which were under normal limits. A follow-up was given after seven days, in which it was seen that there was reduced gingival inflammation. After seven days of completing the initial therapy, the gingivectomy procedure was done using the conventional method. The procedure started with administering local anesthesia followed by making bleeding points. The external bevel incision was given at 45 degrees through the bleeding points with a bevel from the apical and coronal to the mucogingival junction. After this, the pocket lining was removed (Figure 2). After achieving hemostasis, the periopack GC Coe-Pack (GC America Inc., USA) was given for healing and protecting the tissues (Figure 3).

**Figure 4:**
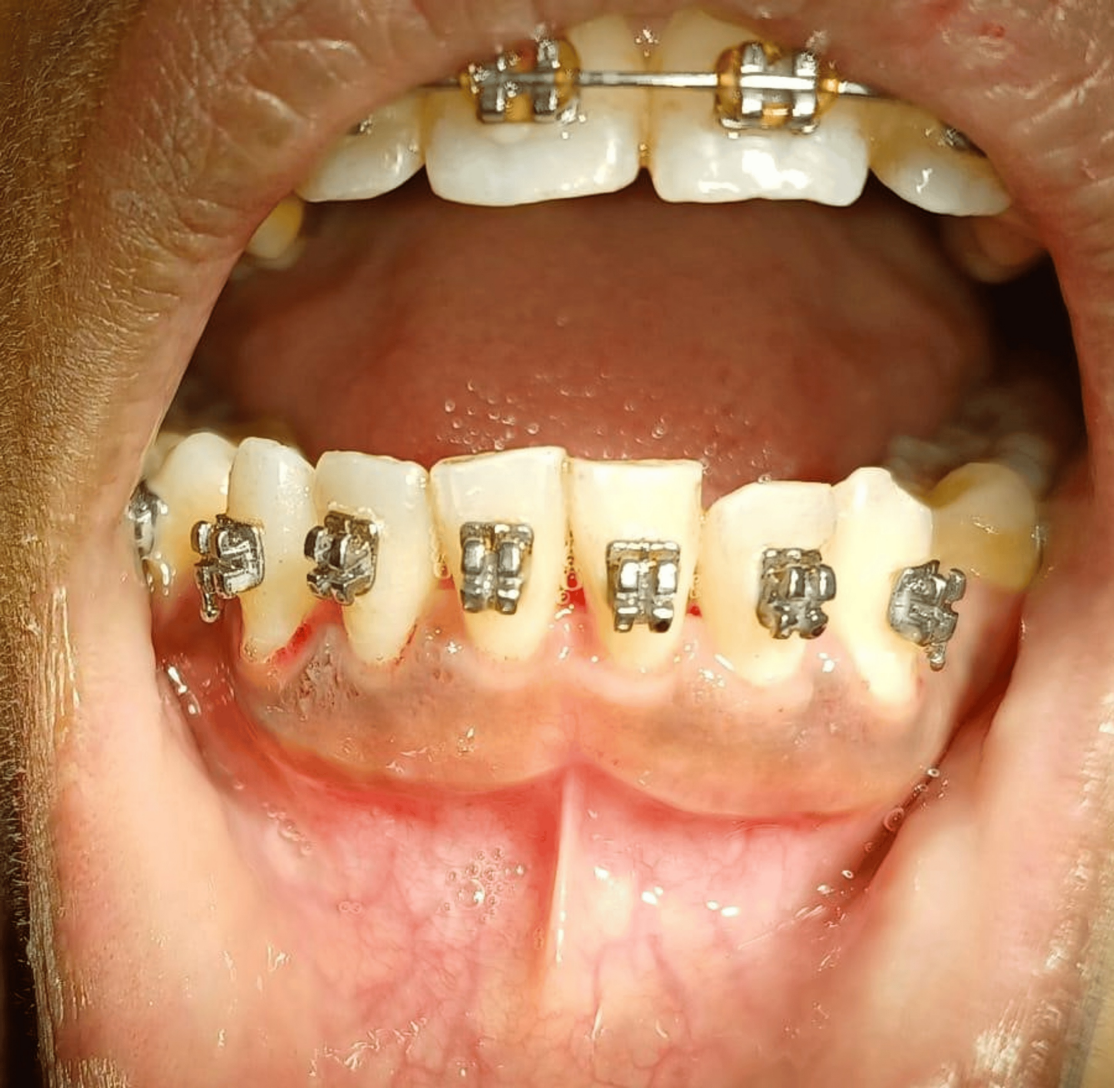
Postoperative image. Postoperative view after seven days revealed satisfactory healing, proper gingival contour, and improved esthetics. Image credit: Komal Agrawal

Analgesics and antiseptic mouth rinse were prescribed. A recall after seven days was given for the removal of periodontal dressing. The gingival contour was found normal and satisfactory healing was seen. A follow-up after two months was given in which it was noticed that proper healing had occurred (Figure 4).

## Discussion

It has been determined that one of the variables that contribute to patients' gingival expansion or overgrowth is orthodontic treatment. The type of treatment to be performed in this case is the most important choice. The external bevel gingivectomy was selected due to its ability to achieve the desired gingival profiles as well as remove excess gingival. With this procedure, the surgeon can remove hypertrophic tissue with minimal to no damage to surrounding structures [9]. Surgery continues to be the most common form of treatment for gingival development; this kind of care is used when development is severe. These consist of laser excision, overgrowth flap surgery, scalpel gingivectomy, and electrosurgery [6]. According to Al-Abdaly et al., salivary flow, pH, gingival enlargement index, and predictive values for inflammatory gingival enlargement and its treatment are the effective indices. Therefore, throughout orthodontic therapy, these findings can be used to monitor the degree of inflammation associated with gingival enlargement [9].

In the given case, in the patient, gross gingivitis without any periodontal bone loss was identified and followed by a medical examination that included periodontal probing as well as a radiographic evaluation. Achieving satisfactory hemostasis was necessary because blood loss obstructs tissue removal and visualization. With the aid of local anesthetics that included vasoconstrictors and an additional cautious surgical approach, the issue of hemorrhage development that was observed in the past was effectively addressed. Another crucial aspect involved monitoring the patients' adherence to the post-operative care instructions, which is important for prompt recovery and to prevent recurrence [4]. The most prevalent illness treated using external bevel surgery, which requires making incisions in the free gingiva, is gingival surgery expansion. The formation of an open wound on the gingiva's surface is a drawback of this treatment. The periodontal pack is necessary to prevent bacterial load and lower the risk of blood clot formation following gingivectomy [5]. The evaluation conducted on the intervention's short-term results demonstrated it had a positive impact on patients' comfort and gingival form. The patient reported great satisfaction with the functional and aesthetic outcome, and the healing process proceeded without any complications. This case highlights the need for appropriate education and prior planning as indicators of successful periodontal surgery outcomes.

## Conclusions

This case report provides proof that gingivectomy is an effective treatment because it fixes the issue entirely. After a comprehensive preliminary evaluation, the surgeon took specific actions to enhance the patient's postoperative experience as well as gingival contours through strategic and technical management. The result in this instance supports the significance of meticulous preoperative preparation, skilled supervision in the operating room, and thorough postoperative care. Patient education and compliance are also essential for improving results and preventing the issue from coming up again. As a result, this instance validates the use of gingivectomy in periodontal therapy and adds to the body of knowledge regarding its application.
